# Pyogenic Spondylodiscitis: Predictors of Microbiological Yield from Biopsy in a Tertiary Hospital

**DOI:** 10.3390/medicina61091591

**Published:** 2025-09-03

**Authors:** Aslı Haykır Solay, Dilek Bulut, Gülnur Kul, Semanur Kuzi, Muhammed Erkan Emrahoğlu, İhsaniye Süer Doğan, Nesibe Korkmaz, Ayşenur Soykuvvet Ayhan, Fatma Şanlı, Mustafa Kavcar, Saffet Öztürk, Gönül Çiçek Şentürk

**Affiliations:** 1 Department of Infectious Diseases and Clinical Microbiology, Etlik City Hospital, 06170 Ankara, Türkiyegulnur.kul1@saglik.gov.tr (G.K.); semanur.kuzi@saglik.gov.tr (S.K.);; 2 Department of Neurosurgery, Etlik City Hospital, 06170 Ankara, Türkiye; erkan.emrahoglu@saglik.gov.tr (M.E.E.);; 3 Department of Radiology, Etlik City Hospital, 06170 Ankara, Türkiye

**Keywords:** Pyogenic spondylodiscitis, microbiological yield, tissue biopsy, alkaline phosphatase, serum albumin

## Abstract

*Background and Objectives*: Pyogenic spondylodiscitis (SD) is a severe spinal infection involving the intervertebral disc and adjacent vertebrae and is often associated with significant morbidity. Identifying the causative microorganism is crucial for targeted treatment; however, the microbiological yield from blood or tissue cultures varies widely due to factors such as prior antibiotic use and biopsy technique. In this study, we aimed to investigate the clinical, laboratory, and radiological predictors of microbiological yield, particularly from tissue biopsy specimens. *Materials and Methods*: This retrospective cohort study included adult patients diagnosed with pyogenic SD between January 2023 and July 2025 at a tertiary care hospital. Demographics, comorbidities, laboratory markers (CRP, ESR, ALP, albumin), radiological findings (abscess presence, anatomical location, claw sign), prior antibiotic use, and microbiological results were analyzed. Tissue specimens were obtained through either surgical sampling or needle biopsy. Univariable and multivariable logistic regression were performed to determine the predictors of positive tissue cultures. *Results*: Of the 159 patients screened, 55 met our inclusion criteria. The mean age was 63.9 ± 13.5 years, 80% had lumbar involvement, and 58.2% had abscesses, primarily paravertebral or psoas in location. Microorganisms were isolated in 65.5% of the cases, with Staphylococcus aureus being the most common (41.7%). The blood culture positivity was 55.5%, while tissue culture positivity was 40.4%. Logistic regression revealed that lower albumin (*p* = 0.046) and higher ALP levels (*p* = 0.045) were independent predictors of a positive microbial yield from tissue biopsies. *Conclusions*: Serum albumin and ALP levels may aid clinical decision-making regarding invasive sampling in SD. When blood cultures are negative and albumin is low while ALP is elevated, clinicians should consider prioritizing tissue biopsy. These findings may help optimize diagnostic strategies and should be validated in larger, prospective studies.

## 1. Introduction

Spondylodiscitis (SD) is characterized by infection of the intervertebral disc and adjacent vertebral bodies. Although rare—estimated at 0.4–2.4 cases per 100,000 person-years—it is a serious spinal infection associated with significant morbidity and mortality, often leading to chronic pain, neurological sequelae, and reduced quality of life. The incidence has risen in recent decades, likely due to increased life expectancy, the growing population of immunocompromised patients, and widespread use of spinal instrumentation and invasive procedures. Epidemiological studies consistently demonstrate a higher prevalence among elderly individuals and males, with risk factors including diabetes mellitus, chronic kidney disease, end-stage renal disease requiring hemodialysis, malignancy, and conditions necessitating immunosuppressive therapy. Additional risk factors include intravenous drug use, long-term corticosteroid treatment, and recent spinal surgery or epidural interventions [1,2]. Although the infection usually reaches the vertebral structures via hematogenous spread, it may also occur through direct inoculation (e.g., spinal surgery, epidural interventions) or contiguous spread from adjacent infected tissues [1,2].

The clinical presentation is often nonspecific, typically characterized by back pain, fever, and elevated levels of inflammatory markers. Because these features overlap with those of other spinal disorders, diagnosis is frequently delayed—sometimes by several weeks—which may increase the risk of complications such as vertebral collapse, epidural abscesses, or irreversible neurological deficits [1]. Diagnosis typically relies on clinical evaluation, imaging studies, and laboratory findings. However, identification of the causative microorganism is crucial for initiating targeted and effective antimicrobial therapy. For this purpose, blood cultures, image-guided percutaneous biopsies, and—in selected cases—surgically obtained biopsy specimens are the most commonly used methods for microbiological analysis [3]. Despite their widespread use, image-guided needle biopsies often demonstrate limited sensitivity, especially in culture-negative cases, making diagnosis challenging. This highlights the need for predictive clinical or laboratory markers that can help guide decisions about invasive diagnostic procedures and optimize microbiological yield.

*Staphylococcus aureus* is the predominant pathogen in pyogenic spondylodiscitis (30–65% of cases), followed by enteric Gram-negative bacilli, streptococci, *Enterococcus* spp., and *Pseudomonas aeruginosa*. The distribution of pathogens varies depending on individual patient risk factors [2,3]. Reported microbiological culture positivity rates in pyogenic SD vary widely, ranging from 30% to 70% [1]. This variability depends on several factors, such as the timing of sample collection, initiation of antibiotic therapy, method of tissue access, and quality of the specimen [4]. When blood or tissue cultures fail to identify a causative microorganism, the diagnostic and therapeutic process becomes more challenging and often necessitates prolonged use of empirical broad-spectrum antibiotics.

Therefore, a better understanding of clinical and laboratory parameters that influence microbiological diagnostic success is crucial for facilitating diagnosis and improving treatment outcomes [3,4]. In this study, we evaluated the effects of demographic and clinical characteristics, along with laboratory and imaging findings, on microorganism isolation in patients diagnosed with pyogenic SD at a tertiary care hospital. By identifying conditions under which biopsy leads to a higher rate of pathogen isolation, we aimed to provide evidence to support clinical decision-making regarding the need for invasive diagnostic procedures.

## 2. Materials and Methods

This retrospective cohort study included patients diagnosed with SD and followed in our clinic between January 2023 and July 2025. All patients who met the inclusion criteria were enrolled in this study.

The inclusion criteria for this study included patients aged 18 years or older with radiological findings suggestive of SD on magnetic resonance imaging (MRI) or computed tomography (CT) and one of the following: (a) isolation of a causative microorganism from blood or tissue cultures and/or histopathological findings consistent with SD [4] or, (b) in cases where both culture and histopathology were negative, inclusion was based on clinical and laboratory response to empirical antibiotic therapy. A treatment response was defined as a significant reduction in pain, a decrease in erythrocyte sedimentation rate (ESR) of more than 30%, or to a level below 50 mm/h, and/or a C-reactive protein (CRP) level falling below 2.75 mg/dL one month after the initiation of treatment [5].

Exclusion criteria included patients under 18 years of age, pregnant women, those with polymicrobial growth in blood and/or tissue cultures, cases of SD secondary to tuberculosis or brucellosis, patients who received empirical treatment but showed no clinical or laboratory response, those with non-infectious vertebral involvement due to trauma, malignancy, or rheumatologic diseases, and patients with incomplete follow-up or missing clinical data.

Tuberculous and brucellar SD cases were excluded because their diagnostic and microbiological confirmation methods differ substantially from those of pyogenic cases. Brucella SD is often diagnosed serologically rather than by culturing, limiting comparability in microbiological yield analyses. Tuberculous SD requires specialized culture methods and prolonged incubation, and both infections exhibit distinct epidemiological, pathophysiological, and therapeutic profiles, thereby potentially introducing heterogeneity into a cohort focused on acute pyogenic infections.

### 2.1. Data Collection Method

Data were obtained from the hospital information system and patient medical records. Demographic characteristics such as age and sex were recorded, along with comorbid conditions such as diabetes mellitus, chronic kidney disease, and immunosuppression. Clinical features—such as the duration of symptoms prior to diagnosis, fever, neurological deficits, and a history of prior spinal surgery or procedures—were recorded. Laboratory data included complete blood count, acute phase reactants (CRP, ESR, and procalcitonin), and biochemical parameters [alkaline phosphatase (ALP), albumin, and renal and liver function tests]. Information on prior antibiotic use and its duration was also noted. For patients who underwent tissue culture, the hospital day on which the procedure was performed was recorded.

Radiological evaluations included the anatomical location of involvement (cervical, thoracic, thoracolumbar, or lumbar), vertebral body and/or disc involvement, and the presence of paravertebral or epidural abscess. On diffusion-weighted MRI sequences, the “claw sign” was defined as well-demarcated, linear, and typically symmetrical areas of high signal intensity in the vertebral bodies adjacent to the affected disc level [6]. Presence or absence of the claw sign was recorded in all MRI scans.

Blood cultures were obtained prior to the initiation of antimicrobial therapy whenever feasible. For tissue cultures, percutaneous needle biopsies were performed under CT or fluoroscopic guidance by interventional radiologists using an 11–13-gauge coaxial bone biopsy needle. In cases where percutaneous sampling was nondiagnostic or technically unfeasible, open surgical biopsies were performed by spine surgeons. All samples were processed in the hospital’s microbiology laboratory, inoculated onto standard aerobic and anaerobic culture media, and incubated in accordance with international protocols. Isolates were identified using MALDI-TOF mass spectrometry, while antimicrobial susceptibility testing was conducted using automated systems.

Histopathological specimens were examined for inflammatory cell infiltration, necrosis, and granulation tissue consistent with infectious SD.

### 2.2. Statistical Analysis

Statistical analyses were performed using IBM SPSS Statistics software (version 31, IBM Corp., Armonk, NY, USA). The distribution of continuous variables was assessed with the Shapiro–Wilk test. Non-normally distributed variables were expressed as the median (25–75th percentiles) and compared between groups using the Mann–Whitney U test. Categorical variables were presented as frequency and percentage (%) and compared using the Pearson chi-square test or Fisher’s exact test, where appropriate.

To identify factors associated with pathogen isolation, variables found to be significant in univariate analyses were included in a multivariate logistic regression model. Independent effects of CRP, ESR, albumin, and ALP levels on microorganism isolation were evaluated. Model fit was assessed using the Hosmer–Lemeshow goodness-of-fit test and was found to be adequate (χ^2^(5) = 1.305, *p* = 0.934). Results were reported with B coefficients, standard error (SE), Wald statistics, *p*-values, odds ratios (ORs), and 95% confidence intervals (CIs).

A *p*-value < 0.05 was considered statistically significant for all tests.

This study was approved by the Scientific Research Ethics Committee of Ankara Etlik City Hospital (approval number: AEŞH-BADEK-2025-0063). The study was conducted in accordance with the principles of the Declaration of Helsinki.

## 3. Results

In this retrospective study, the medical records of 155 patients diagnosed with SD and followed in our clinic between January 2023 and July 2025 were reviewed. A total of 34 cases of brucellar SD, 18 of tuberculous SD, 15 of polymicrobial growth in blood and/or tissue cultures, 25 with incomplete data, and eight with no treatment response were excluded from this study. Fifty-five cases of pyogenic SD meeting the inclusion criteria were evaluated.

The mean age of the patients was 63.9 ± 13.5 years (range: 21–84), and 50.9% (*n* = 28) were male. The most common presenting symptoms were pain (90.9%, *n* = 50), fever (47.3%, *n* = 26), and neurological deficits (21.8%, *n* = 12). Secondary SD developed following surgical interventions in 10 patients (18.2%).

The most frequent comorbidities were diabetes mellitus (43.6%, *n* = 24) and chronic kidney disease (21.8%, *n* = 12), while immunosuppression was identified in five patients (9%). Of these five patients, two were undergoing hemodialysis; one had a history of renal transplantation; one had psoriasis; and one had hidradenitis suppurativa and was receiving tumor-necrosis-factor-alpha-inhibitor therapy.

Regarding anatomical involvement, the lumbar vertebrae were affected in 80% (*n* = 44) of cases, and both the vertebral body and disc were involved in 60% (*n* = 33). Abscess formation was observed in 58.2% (*n* = 32) of cases, most commonly in the paravertebral (23.6%, *n* = 13) and psoas (20%, *n* = 11) regions. Epidural abscess was detected in 5.5% (*n* = 3) of patients. The claw sign was positive in 20% (*n* = 11) of cases, and a pathogen was isolated in five of these patients (Table 1).

Blood cultures were obtained from 81.8% (*n* = 45) of cases, and tissue cultures from 76.4% (*n* = 42). Of the tissue samples, 32 were obtained via surgical sampling and 10 via needle biopsy.

A causative microorganism was identified in 36 patients (65.5%). Among these, Gram-positive pathogens were isolated in 23 cases (63.8%). The distribution of pathogens is presented in Table 2.

The pathogen isolation rate from blood cultures was 55.5% (twenty-five of forty-five patients), and tissue cultures yielded positive results for seventeen patients (40.4% of those sampled): twelve from surgical specimens and five from needle biopsies. The isolation rates were 37.5% (12/32) for surgical sampling and 50% (5/10) for needle biopsies. In seven patients, pathogens were identified in both blood and tissue cultures.

Analysis of tissue sample timing revealed that the procedure was performed a mean of 16.95 ± 26.53 days after hospital admission. The median tissue culture analysis time was 6 (range, 1–155) days. This value was 5 (range, 2–20) days in the patient group with tissue culture (+) and 14 (range, 1–155) days in the patient group with tissue culture (−). There was a statistically significant difference between the two groups in terms of the median tissue culture analysis time (*p*: 0.010). Prior antibiotic use was noted in 34.5% (*n* = 19) of patients, with a mean duration of 7.02 ± 11.41 days. Among those who underwent surgical sampling, 11 (34.3%) had received antibiotics prior to the procedure, compared with 5 (50%) among those who underwent needle biopsy. Antibiotic therapy was initiated for patients with neurological deficits for whom rapid clinical deterioration was anticipated and prompt arrangements for needle or surgical biopsy were not feasible.

Histopathological examination was performed in 59.5% (*n* = 25) of patients who underwent tissue sampling. Findings consistent with SD were detected in 19 cases, of which 5 showed no growth in blood and/or tissue cultures.

When comparing patients with and without pathogen isolation, significant differences were observed in certain clinical and laboratory parameters. The mean age of patients with pathogen isolation was higher, with the difference approaching statistical significance (*p* = 0.069). Pathogen isolation was significantly more frequent in patients with fever (*p* = 0.024), whereas prior antibiotic use had no significant effect.

In laboratory analyses, white blood cell (WBC) count, ESR, CRP, procalcitonin, and ALP levels were significantly higher, while albumin levels were significantly lower in the pathogen-positive group (*p* < 0.05) (Table 3). For patients with elevated ALP levels, gamma-glutamyl transferase (GGT) measurements and liver ultrasonography were performed: the results were within normal limits, thereby ruling out cholestatic conditions of the biliary tract. An ALP isoenzyme analysis could not be performed.

When parameters affecting tissue culture positivity were analyzed, biopsy method, prior antibiotic use, duration of antibiotic therapy, and anatomical site of involvement did not differ significantly between groups. However, ESR, CRP, albumin, and ALP levels showed significant differences. Patients with positive tissue cultures had significantly higher ESR (*p* = 0.027) and CRP levels (*p* = 0.021) and significantly lower albumin levels (*p* = 0.004). Additionally, ALP levels and the proportion of patients with ALP ≥ 140 U/L were significantly higher in the culture-positive group (*p* = 0.018 and *p* = 0.041, respectively). Other parameters, such as procalcitonin, WBC, and CRP > 50 mg/L, did not show significant differences (*p* > 0.05) (Table 4).

Regression analysis was performed to evaluate the predictive value of procalcitonin level, WBC count, CRP level, albumin level, ESR, and ALP level with respect to microbial growth. According to the regression model, only albumin and ALP levels were significant predictors of microbial growth. An increase in albumin level significantly reduced the likelihood of microbial growth (B = −3.47, SE = 1.74, Wald = 3.97, *p* = 0.046; odds ratio = 0.031; 95% CI [0.001, 0.945]). Conversely, an increase in ALP level significantly increased the likelihood of microbial growth (B = 3.22, SE = 1.60, Wald = 4.03, *p* = 0.045; odds ratio = 25.02; 95% CI [1.077, 581.05]). Neither CRP (B = 18.23, *p* = 0.999) nor ESR (B = 0.75, *p* = 0.617) was a significant predictor of microbial growth.

## 4. Discussion

In this study, we investigated clinical, laboratory, and radiological factors associated with microbiological yield in patients with pyogenic SD, focusing particularly on tissue cultures. Our findings revealed that, while neither the biopsy method nor prior antibiotic use significantly affected culture positivity, lower serum albumin and higher ALP levels were independently associated with microbial growth from tissue specimens. Notably, although surgical biopsies are generally considered more reliable, needle biopsies in our cohort demonstrated a comparable—and slightly higher—positivity rate, likely due to sample size limitations. Furthermore, histopathological analysis contributed meaningfully to the diagnosis in a subset of culture-negative cases, underscoring its complementary diagnostic role. These results highlight the potential utility of simple biochemical markers in guiding invasive diagnostic decisions, particularly when conventional culture methods are inconclusive.

Since the early 2000s, a notable global increase in the incidence of pyogenic SD has been reported, with growth rates ranging from 44% to 104%. This rise is attributed to several factors, including a growing elderly population and the increasing prevalence of immunocompromised individuals. Consequently, the healthcare burden has intensified in terms of both costs and clinical resources [7,8,9,10]. This trend highlights the need for heightened clinical vigilance and more effective diagnostic strategies. In our study, we evaluated 55 cases of pyogenic SD managed at a single center over a 2.5-year period. The combined assessment of clinical, microbiological, and radiological findings provides a more comprehensive diagnostic perspective.

The increase in SD incidence has also been linked to broader use of intravascular devices, which elevate the risk of bacteremia, and to the growing prevalence of spinal instrumentation procedures. Additional contributing factors include the aging population, increased number of patients undergoing renal replacement therapy, wider use of immunosuppressive therapies, and more frequent application of spinal interventions [1,7]. Furthermore, healthcare-associated hematogenous SD accounts for approximately one-third of all pyogenic SD cases, rendering it a significant public health concern in terms of preventable nosocomial infections [9]. In our cohort, the mean age was 63.9 ± 13.5 years, consistent with the elderly predominance reported in the literature. Secondary SD following surgical intervention was observed in 18.2% of patients. Common comorbidities included diabetes mellitus (43.6%), chronic kidney disease (21.8%), and immunosuppression (9%).

Pain is the most frequently reported symptom in SD, occurring in approximately 86% of cases, while fever is observed in only about half of patients. Neurological deficits, including sensory loss, muscle weakness, or radiculopathy, are reported in roughly one-third of cases [1]. In our study, pain was the most common presenting complaint (90.9%), followed by fever (47.3%) and neurological deficits (21.8%).

Anatomically, the lumbar vertebrae are most commonly affected in SD, followed by the thoracic and cervical regions [1]. The presence of abscesses, indicative of infection spread, is influenced by the extent of vertebral involvement, regional adipose tissue, and structural characteristics of the epidural space. This is particularly relevant in elderly patients and those undergoing frequent invasive procedures [9,10,11]. In our study, lumbar involvement was noted in 80% of cases. Abscesses were present in 58.2% of cases, most frequently in the paravertebral (23.6%) and psoas (20%) regions, while epidural abscesses were less common (5.5%).

The claw sign is radiologically defined as a well-demarcated, linear, and typically bilateral area of high signal intensity in the vertebral bodies adjacent to the affected disc level. It is generally associated with non-infectious etiologies in up to 97–100% of cases and has been proposed to be a feature that helps exclude pyogenic SD [5]. However, in our study, the claw sign was observed in 20% of patients (*n* = 11), and a causative microorganism was isolated in five of these cases. This suggests that the presence of the claw sign alone is insufficient to rule out SD and should be interpreted cautiously.

*Staphylococcus aureus* is the most frequently isolated organism in pyogenic SD, accounting for 30–65% of cases. Less frequently encountered organisms include enteric Gram-negative bacilli, viridans group streptococci, group B/C/G streptococci, *Enterococcus* spp., and *Pseudomonas aeruginosa*. The microbial spectrum may vary depending on the underlying risk profile of the patient [1,2]. Moreover, coagulase-negative staphylococci (CoNS) are more commonly detected in post-surgical infections [1,2]. In our cohort, the most frequently isolated pathogen was S. aureus (41.7%), followed by CoNS (19.5%), Escherichia coli and Klebsiella spp. (16.7% each), and Pseudomonas spp. and Streptococcus spp. (2.8% each). This distribution is largely consistent with previously reported patterns.

Microbiological confirmation is critical in guiding targeted treatment. Blood cultures have reported positivity rates ranging from 30% to 78% [1,3]. When cultures are negative or a polymicrobial infection is suspected, tissue biopsy is recommended [12]. Image-guided needle biopsies are commonly used, with reported sensitivities between 31% and 91% [4,12,13]. A meta-analysis by McNamara et al. (*n* = 1763 patients) reported a detection rate of 48% for image-guided biopsies and 76% for surgical biopsies [14]. In our study, blood cultures were positive in 55.5% of cases and tissue cultures in 40.4%. Interestingly, the yield was higher with needle biopsy (50%) compared with surgical sampling (37.5%), which likely reflects sample size limitations.

Histopathological analysis is particularly useful when cultures are negative. Tissue samples should ideally be evaluated both microbiologically and histologically. Relevant histological features include chronic inflammation, granulomatous response, necrosis, and the presence of microorganisms [15]. In our cohort, only 59.5% of tissue-sampled patients underwent histopathological analysis. In five culture-negative cases, diagnosis was achieved solely through histopathological findings, underscoring its diagnostic value.

Several studies have investigated factors associated with culture positivity. The impact of prior antibiotic use remains debated. McNamara et al. suggested a possible reduction in yield, but their meta-analysis did not confirm a statistically significant effect [14]. In most studies, the effect of the timing of tissue culture sampling on culture yield is evaluated indirectly through prior antibiotic exposure [14,16]. Fragío Gil et al. analyzed timing as an independent variable and found that it did not influence culture yield significantly [17]. Ang et al. found that elevated CRP and leukocytosis were predictors of culture positivity and that abscess aspiration had the highest predictive value [18]. Mutlu et al. identified elevated CRP and ESR, along with paravertebral signal changes on MRI, as associated with culture positivity [19]. In our study, factors such as age, sex, abscess presence, anatomical site, comorbidities, and antibiotic use or duration did not significantly affect pathogen identification. In contrast, our study demonstrated that earlier tissue sampling was associated with an increased culture yield. However, fever, leukocytosis, elevated CRP, ESR, procalcitonin, and ALP, along with decreased albumin, were significantly associated with pathogen isolation. Subgroup analysis of tissue culture cases yielded similar results: higher ESR, CRP, and ALP, and lower albumin levels in culture-positive patients. Regression analysis confirmed low albumin and elevated ALP as independent predictors of microbial growth in tissue cultures. To the best of our knowledge, no prior study has identified these biochemical parameters as independent predictors of microbiological yield from biopsy, which represents a novel contribution of this study.

ALP is an enzyme predominantly expressed in the liver, bone, intestinal mucosa, and placenta, with liver- and bone-derived isoenzymes contributing most to serum levels in adults. Elevated ALP levels may occur in various conditions, including cholestasis, biliary tract obstruction, and systemic inflammatory states; however, in the context of bone metabolism, increased ALP reflects enhanced osteoblastic activity, leading to elevated levels of inorganic phosphate in the extracellular matrix and the subsequent promotion of mineralization [20,21]. In our study, patients with elevated ALP levels were evaluated for cholestasis and biliary tract obstruction, but none were found to have these conditions. Therefore, the elevation of ALP was considered to be of bone origin. ALP elevation is not a common finding in osteomyelitis cases. In the study by Pääkkönen et al., which evaluated ALP levels in pediatric osteomyelitis, the increase in ALP levels was attributed to osteoblastic activity during the healing phase [22]. In cases of SD, ALP elevation is likely associated with increased osteoblastic activity in response to infection-induced bone destruction. Although no studies have directly explained this mechanism, several potential pathophysiological processes can be proposed. Accelerated bone turnover may reflect a more extensive and active infection, which could indicate a higher bacterial load within the infected tissue, thereby increasing the likelihood of culture positivity from biopsy specimens. Moreover, angiogenesis triggered by active inflammation may facilitate a more homogeneous distribution of microorganisms within the lesion, thus enhancing the success of microbiological diagnosis.

In cases of SD, the causative microorganisms may include pyogenic bacteria, *Mycobacterium tuberculosis*, or *Brucella* spp. While brucellar SD can often be diagnosed through serological tests and imaging modalities, culture-based methods are typically required to confirm the diagnosis in pyogenic and tuberculous cases. In certain pyogenic infections (e.g., with *S. aureus* and *S. lugdunensis*), the isolation of the organism from blood cultures may be considered sufficient for diagnosis; however, in a subset of cases, tissue biopsy for culture remains necessary [6].

**Figure 1:**
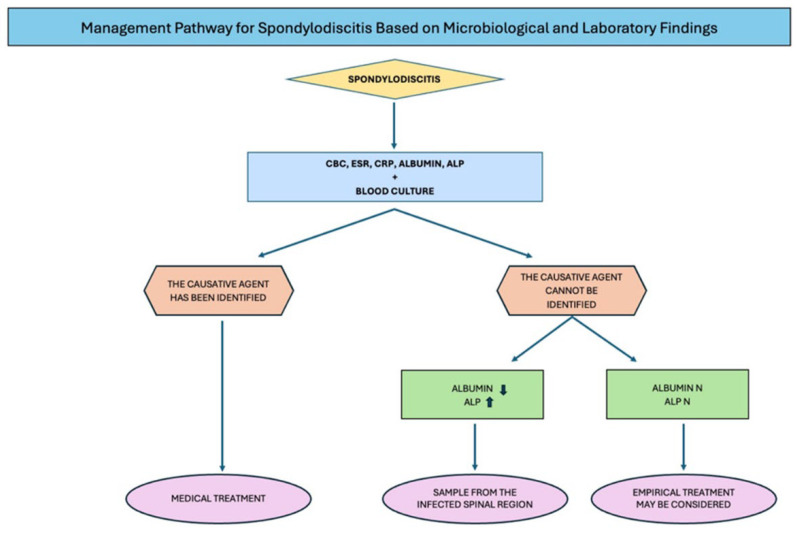
Management of spondylodiscitis.

Nevertheless, performing these cultures is not feasible in many centers, and it may be a time-consuming process, leading to delays in initiating appropriate treatment and increasing the risk of complications. Therefore, early microbiological investigation through tissue biopsy is crucial for pathogen identification and the timely initiation of a targeted antimicrobial therapy. Tissue samples may be obtained via a computed tomography (CT)-guided needle biopsy or a surgical biopsy. The choice of method depends on the patient’s clinical status, their comorbidities, and the technical resources available at the healthcare facility in question [23,24]. Invasive procedures may not be available in all centers nor applicable to every patient. This study highlights the importance of determining when to insist on invasive procedures for microbiological diagnosis in suspected pyogenic SD cases (Figure 1).

This study has several limitations. First, its retrospective and single-center nature limits generalizability. Second, its relatively small sample size may have reduced statistical power, especially in subgroup analyses. Third, culture acquisition was based on clinical judgment, introducing heterogeneity in sampling. Fourth, histopathological analysis was performed in only 60% of tissue-sampled patients, potentially underestimating its diagnostic contribution. Finally, procedural and operator-dependent variations in imaging and biopsy techniques during the study period may have influenced its outcomes.

## 5. Conclusions

Based on these findings, blood cultures should be obtained from all patients with SD. However, obtaining tissue cultures may not be feasible in every clinical setting due to resource constraints or patient comorbidities that limit the safety of invasive procedures. In such scenarios, the combined evaluation of serum albumin and ALP levels may assist in clinical decision-making regarding the necessity of tissue biopsy. Specifically, greater efforts should be made to obtain tissue samples in patients with low albumin and high ALP levels.

These results suggest that certain laboratory parameters may support clinical prediction and guide decisions concerning invasive diagnostic procedures in patients with SD. Given the essential role of microbiological confirmation in directing appropriate therapy, the improved identification of patient profiles more likely to yield positive cultures may enhance diagnostic efficiency and patient outcomes. To validate these findings, further prospective, multi-center studies with larger sample sizes are warranted.

## Figures and Tables

**Table 1:** Distribution of anatomical involvement and radiological findings in patients with spondylodiscitis.

	*n* = 55 *n* (%)
Anatomical Involvement Region	
Cervical	1 (1.8%)
Thoracic	10 (18.2%)
Thoracolumbar	4 (7.3%)
Lumbar	30 (54.5%)
Lumbosacral	10 (18.2%)
Pattern of Involvement	
Vertebral Body	5 (9.1%)
Intervertebral Disc	17 (30.9%)
Vertebral Body and Disc	33 (60%)
Claw Sign	11 (20%)
Presence of Abscess	32 (58.2%)
Abscess Location	
Psoas	11 (34.4%)
Paravertebral	13 (40.6%)
Epidural	3 (9.4%)
Other	5 (5.6%)

**Table 2:** Microorganism distribution (grouped by species, ordered by frequency).

Microorganism Type	*n* (%)
*Staphylococcus aureus*	15 (41.7%)
Coagulase-negative staphylococci (CoNS)	7 (19.5%)
*Escherichia coli*	6 (16.7%)
*Klebsiella* spp.	6 (16.7%)
*Pseudomonas* spp.	1 (2.8%)
*Streptococcus* spp.	1 (2.8%)
Total Positive Isolations	36 (100%)

**Table 3:** Comparison of clinical and laboratory parameters between culture-positive and culture-negative patients.

Parameter	Culture (+) (*n* = 36)	Culture (−) (*n* = 19)	*p*
Age (mean ± SD)	65.8 ± 13.9	60.5 ± 12.2	0.069 ^c^
>65 years	24 (66.7%)	8 (42.1%)	0.079 ^b^
Fever	21 (58.3%)	5 (26.3%)	**0.024** ^a^
Abscess presence	24 (66.7%)	8 (42.1%)	0.079 ^a^
Diabetes mellitus	17 (47.2%)	7 (36.8%)	0.460 ^a^
Chronic kidney disease	10 (27.8%)	2 (10.5%)	0.183 ^b^
History of spinal surgery	5 (13.9%)	5 (26.3%)	0.256 ^a^
WBC (median, range)	10,440 (3580–35,690)	8410 (2550–13,460)	**0.041** ^c^
WBC > 10,000	20 (55.6%)	6 (31.6%)	0.09 ^a^
Sedimentation (mm/h)	62.5 (12–140)	38 (4–128)	**0.002** ^c^
Sedimentation > 50 mm/h	28 (77.8%)	7 (36.8%)	**0.003** ^a^
CRP (mg/L)	159 (8–528)	30 (2–130)	**<0.001** ^c^
CRP > 50 mg/L	31 (86.1%)	7 (36.8%)	**<0.001** ^b^
Procalcitonin (ng/mL)	0.475 (0.05–97)	0.080 (0.03–12.6)	**0.005** ^c^
Procalcitonin > 0.05	35 (97.2%)	14 (73.7%)	**0.015** ^b^
Albumin (g/L)	30.6 (18.5–45.1)	37.3 (24–44)	**0.001** ^c^
Albumin ≤ 30 g/L	16 (44.4%)	1 (10.5%)	**0.001** ^b^
ALP (U/L)	118 (66–418)	112 (64–311)	0.280 ^c^
ALP ≥ 140 U/L	14 (38.9%)	2 (10.5%)	**0.033** ^b^
Prior antibiotic use	16 (44.4%)	3 (15.8%)	0.083 ^b^
Duration of antibiotic use (days)	16.5 (1–42)	7 (6–7)	0.093 ^c^

^a^: Pearson’s chi-square test, ^b^: Fisher’s exact test, ^c^: Mann–Whitney U test; WBC: white blood cell count, CRP: C-reactive protein, ALP: alkaline phosphatase. Bold values indicate statistically significant results (*p* < 0.05).

**Table 4:** Tissue culture evaluation.

Variable	Tissue Culture (+) (*n* = 17)	Tissue Culture (−) (*n* = 25)	*p*
Biopsy Method			
Surgical Biopsy	12 (70.6%)	20 (80%)	<0.482 ^a^
Needle Biopsy	5 (29.4%)	5 (20%)	
Antibiotic Use	7 (41.2%)	11 (44%)	0.856 ^c^
Antibiotic Duration (days)	9 (1–37)	15 (6–42)	0.551 ^c^
Anatomical Involvement			
Corpus Only	2 (11.8%)	2 (8%)	0.916 ^a^
Disc Only	5 (29.4%)	8 (32%)	
Corpus and Disc	10 (58.8%)	15 (60%)	
Laboratory Parameters			
WBC (cells/µL)	10,930 (3580–35,690)	9990 (2550–18,330)	0.224 ^c^
WBC (>10,000)	9 (52.9%)	12 (48%)	0.753 ^a^
Sedimentation (mm/h)	72 (21–140)	53 (5–128)	**0.027** ^c^
Sedimentation (>50)	14 (82.4%)	13 (52%)	0.056 ^b^
CRP (mg/L)	139 (8–528)	68 (3–430)	**0.021** ^c^
CRP (>5)	17 (100%)	23 (92%)	0.506 ^b^
CRP (>50)	14 (82.4%)	15 (60%)	0.179 ^b^
Procalcitonin (ng/mL)	0.150 (0.05–97)	0.220(0.03–38.7)	0.980 ^c^
Procalcitonin (>0.05)	16 (94.1%)	22 (88%)	0.635 ^b^
Albumin (g/dL)	30.4 (22–45.1)	35.9 (24–42)	**0.004** ^c^
Albumin (≤30)	8 (47.1%)	4 (16%)	**0.041** ^b^
ALP (U/L)	162 (77–301)	111 (66–331)	**0.018** ^c^
ALP (≥140)	10 (58.8%)	2 (8%)	**<0.001** ^b^

^a^: Pearson’s chi-square test, ^b^: Fisher’s exact test, ^c^: Mann–Whitney U test; WBC: white blood cell count, CRP: C-reactive protein, ALP: alkaline phosphatase. Bold values indicate statistically significant results (*p* < 0.05).

## Data Availability

The data that support the findings of this study are available from the corresponding author upon reasonable request.

## Abbreviations

The following abbreviations are used in this manuscript:

Abbreviation	Full Form
SD	Spondylodiscitis
MRI	Magnetic Resonance Imaging
CT	Computed Tomography
CRP	C-Reactive Protein
ESR	Erythrocyte Sedimentation Rate
ALP	Alkaline Phosphatase
WBC	White Blood Cell
SPSS	Statistical Package for the Social Sciences
OR	Odds Ratio
CI	Confidence Interval
